# Case report: Apatinib combined with neoadjuvant therapy for primary squamous cell carcinoma of the breast: a case report

**DOI:** 10.3389/fphar.2023.1115422

**Published:** 2023-05-11

**Authors:** Fangfang Gao, Jingtai Li, Haoran Liao, Pingming Fan, Minjian Wang, Yu Liu, Linwei Ding, Guankui Du

**Affiliations:** ^1^ Department of Breast Surgery, The First Affiliated Hospital of Hainan Medical University, Haikou, China; ^2^ Guangxi Medical University Cancer Hospital, Nanning, China; ^3^ Department of Pathology, The First Affiliated Hospital of Hainan Medical University, Haikou, China; ^4^ Key Laboratory of Molecular Biology, Hainan Medical University, Haikou, China; ^5^ Department of Biochemistry and Molecular Biology, Hainan Medical University, Haikou, China; ^6^ Biotechnology and Biochemistry Laboratory, Hainan Medical University, Haikou, China

**Keywords:** primary squamous cell carcinoma of the breast, apatinib, VEGFR, targeted therapy, neoadjuvant

## Abstract

Primary squamous cell carcinoma of the breast is a rare subtype of carcinoma of chemosis for which there is no effective chemotherapy regimen. Breast squamous cell carcinoma is usually “triple negative”, with poor chemotherapy effects and poor prognosis. Here, we report a successful case of primary breast squamous cell carcinoma treated with apatinib. The patient was treated with 2 cycles of apatinib. The efficacy was evaluated as partial remission, and a sublesion of approximately 4 cm fell off.

## Introduction

Primary squamous cell carcinoma (PSCC) of the breast is a very rare subtype of saprophytic carcinoma; it accounts for approximately 0.06%–0.2% of all breast cancers (Chen et al., 2022; Wu et al., 2022). PSCC has large tumors, rapid progression, frequent recurrence, and high mortality (Anne et al., 2019; Shrestha et al., 2021). Surgery and radiotherapy can effectively control low-grade squamous cell carcinoma locally (Qi et al., 2021; Wang et al., 2021). However, the disease recurs at a distant site in approximately 46% of these patients (Wang et al., 2021). Therefore, PSCC requires novel treatment options.

Apatinib is a novel small-molecule tyrosine kinase inhibitor that selectively inhibits VEGFR-2, the primary signaling mediator of VEGF-induced angiogenesis (Wang et al., 2022). Apatinib can inhibit the migration and proliferation of endothelial cells promoted by vascular endothelial growth factor and reduce tumor microvessel density, thereby inhibiting tumor angiogenesis (Feng et al., 2021; Zhang et al., 2022a). Apatinib has been used in the third-line treatment of patients with metastatic gastric cancer; that is, the use of apatinib can effectively improve the progression-free survival and total survival of patients with metastatic gastric cancer who have failed to receive more than two chemotherapy regimens (Yang et al., 2022; Zheng et al., 2022). In addition, Chen et al. demonstrated that the addition of oral apatinib to conventional chemotherapy regimens prolonged progression-free survival in patients with advanced triple-negative breast cancer (Chen et al., 2021). The present report describes the clinical experience with apatinib in a patient with locally advanced PSCC who was insensitive to chemotherapy.

## Case report

In October 2017, a 44-year-old premenopausal woman was admitted to the hospital 6 months after self-discovery of a left breast mass. The patient’s left breast mass was approximately 2 × 2 cm in size when the mass was discovered, and the mass gradually increased in size. The patient scratched the skin due to itching 1 month prior, resulting in localized skin breakdown and oozing on the surface of the lump with a dark green exudate. Due to financial constraints, the patient received local herbal medicine treatment (details are unknown) before admission, but it had no effect. The patient had no family history of a malignant tumor and had not undergone genetic testing.

**FIGURE 1:**
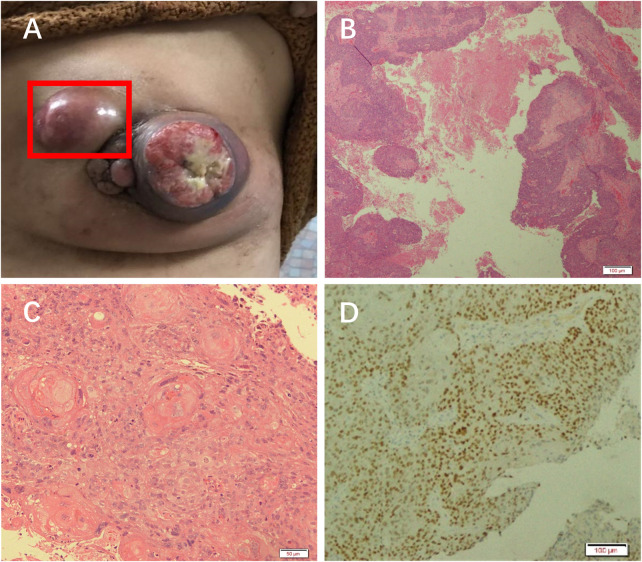
Patient was diagnosed with PSCC. **(A)** A mass in the left breast. H&E staining results at **(B)** 10× and **(C)** ×20 objective. **(D)** Immunohistochemical analysis of GATA.

On examination, a mass was found in the central region of the left breast in the direction of 3 o’clock, with a size of approximately 7 × 6 cm. It was hard, with poorly defined borders, poor mobility, and no tenderness (Figure 1A). The central region of the mass was crater-like, with a large internal cavity and a large amount of necrotic tissue. The swelling was accompanied by two satellite foci, approximately 2 and 4 cm in size. Several enlarged lymph nodes could be detected in the left armpit, the largest of which was located in the pectoralis muscle group. It was approximately 3 × 3 cm in size and hard, with indistinct borders, fusion, poor mobility, and no tenderness. The results of the color ultrasound showed multiple structurally abnormal lymph nodes in the bilateral neck, supraclavicular fossa, and axilla.

The necrotic tissue of the tumor was taken for pathological analysis. H&E staining showed squamous cell carcinoma (Figures 1B, C). Immunohistochemical results showed GATA (+) (Figure 1D), ER (weak-moderate +, approximately 5%), PR (−), Her-2 (1+), Ki-67 (50%), P63 (+), CK5/6 (+), Mammaglobin (−), and GCDFP_15 (−). These results indicated that the patient had a chemosquamous carcinoma of the breast.

In addition, abnormal cells were found in the right supraclavicular lymph node by fine needle biopsy. Immunohistochemical results showed ER(+), PR(+), P63(few+), CK5/6(−), and CK7(+). These results indicated that the patient had metastatic cancer of breast origin. The clinical stage was cT4N2M1.

**FIGURE 2:**
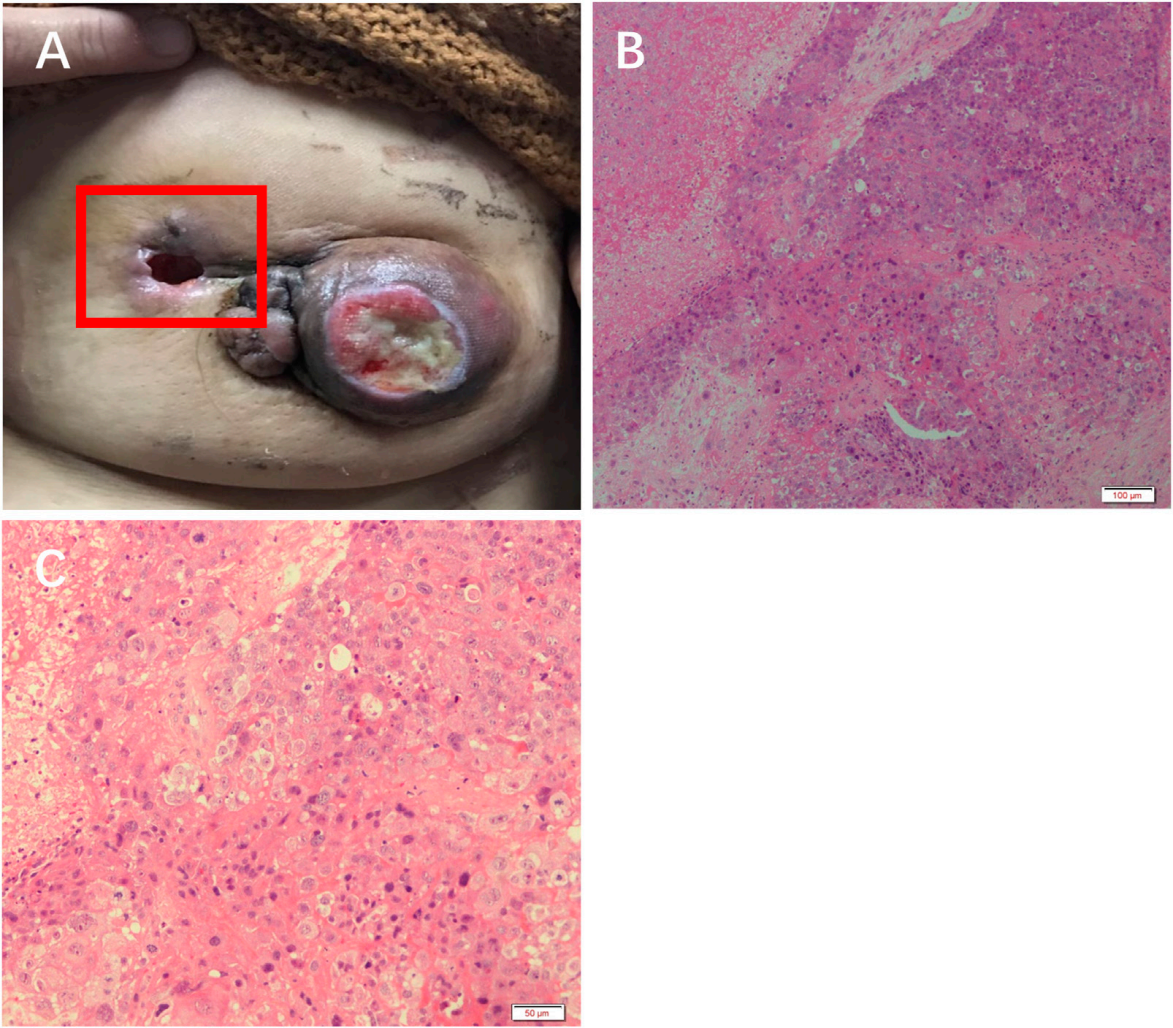
Efficacy of apatinib in this patient. **(A)** The left breast mass fell off. H&E staining results at **(B)** 10× and **(C)** ×20 objective.

The patient received 2 cycles of neoadjuvant chemotherapy with TEC (docetaxel/epirubicin/cyclophosphamide) with an efficacy evaluation of stable disease. Therefore, the treatment regimen was changed to 2 cycles of chemotherapy with Apatinib + TEC (docetaxel/epirubicin/cyclophosphamide) with an efficacy evaluation of PR, and one of the patient’s approximately 4 cm sublesions fell off (Figure 2A).

H&E staining showed massive necrosis of tumor cells, and some residual tumor cells were deeply stained and degenerated after treatment with apatinib (Figure 2B, C).

However, the patient’s poor financial situation prevented her from returning to the hospital on time, and she died after 1 year of treatment.

## Discussion

PSCC is highly heterogeneous and is characterized by the predominance of squamous cells, spindle cells, and (or) mesenchymal metaplasia in invasive cancer, while no adenocarcinoma component can be found (Goel et al., 2021). In addition, the diagnosis of PSCC should include the following features: 1) the absence of other tumor components in the tumor tissue and exclusion of adenocarcinoma to squamous carcinoma; 2) the presence of typical squamous cell carcinoma structure, i.e., intercellular bridges and/or keratinization; 3) exclusion of cancerous tissue originating from the skin; and 4) exclusion of the presence of primary squamous cell carcinoma in other organs or tissues of the body (Goel et al., 2021). In this case, the pathology of this patient showed heterogeneous proliferating cells distributed in nested clusters, with visible angular flower beads and intracellular keratinization. Immunohistochemical results showed GATA (+), which suggested squamous cell carcinoma. Systemic physical examination and instrument examination excluded the occurrence of squamous cell carcinomas, such as skin cancer and esophageal cancer. These pathological features indicate that the patient might have had PSCC.

PSCC has no standard treatment (Yoneto et al., 2018; Alan et al., 2019; Cao et al., 2019; Lei and Miao, 2020). Squamous cell carcinoma of the breast is resistant to standard chemotherapy with IDC (cyclophosphamide, methotrexate, 5-FU, and adriamycin) (Chen et al., 2022). Reports suggest that cisplatin-based chemotherapy regimens are effective for PSCC of the breast (Tsung, 2012; Liu et al., 2015). It has been reported that neoadjuvant chemotherapy, including docetaxel, epirubicin, and cyclophosphamide, can achieve complete pathological remission (Seddik et al., 2015; Alan et al., 2019). In this case, the patient was treated with TEC + apatinib, which alleviated the condition even when the mass fell off. Thus, neoadjuvant chemotherapy plus Apatinib is a novel choice for the treatment of PSCC.

Recent studies have demonstrated the antitumor activity of apatinib in several solid tumors (Zhang et al., 2022b; Xiang et al., 2022; Zhu et al., 2022). Tumor angiogenesis plays an important role in tumor development and metastasis, and antiangiogenic drugs may improve the efficacy of conventional chemotherapy in the treatment of breast cancer (Liu et al., 2021; Lv et al., 2021). Apatinib inactivates the NF-κB signaling pathway, which makes TNBC cells sensitive to anthracyclines *in vitro* and *in vivo*. This provides a sufficient theoretical basis for the combined use of apatinib and anthracyclines in the chemotherapeutic strategy for breast squamous cell carcinoma (Tang et al., 2020). In addition, apatinib can prevent the multidrug resistance conferred by ABCB1 and ABCG2 proteins (Zhang et al., 2019). Apatinib has been successfully used in a variety of tumors that have experienced failure of standard chemotherapy, showing curable results and significant survival benefits (Zeng et al., 2022). Our patient with PSCC showed no progression after 2 rounds of neoadjuvant therapy and showed good remission with the application of apatinib. Therefore, TEC + apatinib may be considered a conventional PSCC treatment.

## Conclusion

This is one of the few reports of the use of apatinib for locally advanced squamous cell carcinoma of the breast with an efficacy assessment of partial remission (PR). Apatinib may be a safe and effective oral targeted agent for patients with locally advanced squamous cell carcinoma of the breast, especially those who have experienced chemotherapy failure or are in poor health.

## Data Availability

The original contributions presented in the study are included in the article/Supplementary Material, further inquiries can be directed to the corresponding authors.
